# Suicidal behaviors in the elderly. About a case

**DOI:** 10.1192/j.eurpsy.2021.1557

**Published:** 2021-08-13

**Authors:** M. ValverDe Barea, M.O. Solis, C. Mata Castro, F. Cartas Moreno

**Affiliations:** Unit Mental Health, Complejo Hospitalario Jaén, Jaén, Spain

**Keywords:** Suicidal Behaviors, major depressive disorder, the elderly, Suicide

## Abstract

**Introduction:**

Suicide is a global health problem. The elderly is the range with the highest suicide rate and suicidal behaviors are more lethal, with greater planning and less possibility of rescue. In the elderly, Major Depressive Disorder is the diagnosis most frequently associated with suicidal behavior. 15% of the elderly with a depressive picture commit suicide. Loneliness, the main cause of suicides in the elderly population.

**Objectives:**

The objective of the clinical case presented is to address the risk factors for suicide in the elderly.

**Methods:**

80-year-old patient, widower who makes a suicide attempt by ingesting glyphosate. Personal history: Acute myocardial infarction 1 month ago. Not mental illness. Family stressors: illness of his granddaughter, loss of his son’s job. Personal stressors: Loss of autonomy due to ischemic heart disease. The patient was admitted to the Intensive Care Unit with acute pulmonary edema secondary to the suicide attempt. Psychopathological exploration: Conscious, oriented and collaborative. Depressive mood in relation to the stressors presented. Makes partial criticism of the suicide attempt, recognizes its seriousness and planning.

**Results:**

Diagnosis: Moderate depressive episode. SAD PERSONS scale: 9 High risk.

**Conclusions:**

The risk factors for suicide in older people can be medical, psychiatric, psychological, family environment and social - environmental factors. There are hardly any specific action protocols that allow early intervention and suicide prevention in the elderly. As social health professionals, we must work on the elaboration and application of these, since consummated suicide represents a major public health problem throughout the world.

